# Wound Healing, Metabolite Profiling, and In Silico Studies of *Aspergillus terreus*

**DOI:** 10.3390/cimb46100694

**Published:** 2024-10-19

**Authors:** Amal A. Al Mousa, Mohamed E. Abouelela, Ahmed Mansour, Mohamed Nasr, Yasser H. Ali, Nadaa S. Al Ghamidi, Youssef Abo-Dahab, Hassan Mohamed, Nageh F. Abo-Dahab, Abdallah M. A. Hassane

**Affiliations:** 1 Department of Botany and Microbiology, College of Science, King Saud University, P.O. Box 145111, Riyadh 4545, Saudi Arabia; nalgamdi1@ksu.edu.sa; 2 Department of Pharmacognosy, Faculty of Pharmacy (Boys), Al-Azhar University, Cairo P.O. Box 11884, Egypt; 3 Department of Pharmacology and Toxicology, Faculty of Pharmacy (Boys), Al-Azhar University, Cairo P.O. Box 11884, Egypt; dr.ahmedmmansour@azhar.edu.eg; 4 Histology Department, Faculty of Medicine, Al-Azhar University, Cairo P.O. Box 11884, Egypt; MohamedNasr2394.el@azhar.edu.eg; 5 Department of Plastic & Reconstructive Surgery, Faculty of Medicine, Al-Azhar University, Cairo P.O. Box 11884, Egypt; dryasserhelmy@azhar.edu.eg; 6 Bioengineering and Therapeutic Sciences Department, University of California, P.O. Box 2520, San Francisco, CA 94158, USA; youssef.abo-dahab@ucsf.edu; 7 Botany and Microbiology Department, Faculty of Science, Al-Azhar University, Assiut P.O. Box 71524, Egypt; hassanmohamed85@azhar.edu.eg (H.M.); abodahabn@azhar.edu.eg (N.F.A.-D.); abdallahhassane@azhar.edu.eg (A.M.A.H.); 8 Colin Ratledge Center of Microbial Lipids, School of Agriculture Engineering and Food Science, Shandong University of Technology, Zibo 255000, China

**Keywords:** *Aspergillus terreus*, wound healing, LC-ESI-MS/MS, molecular docking

## Abstract

Burn injuries, which significantly affect global public health, require effective treatment strategies tailored to varying severity. Fungi are considered a sustainable, easily propagated source for lead therapeutic discovery. In this study, we explored the burn wound healing potential of *Aspergillus terreus* through a combination of in vitro, in vivo, metabolite profiling, and in silico analysis. The in vitro scratch assays performed with human skin fibroblast cells showed promising wound healing activity. Furthermore, the burn-induced rats model showed a marked improvement in cutaneous wound healing, evidenced by an accelerated rate of wound closure and better skin regeneration after *A. terreus* extract treatment at 14 days. The results of this study demonstrated significant enhancements in wound closure and tissue regeneration in the treated rat model, surpassing the outcomes of standard treatments. This controlled healing process, evidenced by superior collagen synthesis and angiogenesis and confirmed by histopathological studies, suggests that *A. terreus* has potential beyond the traditionally studied fungal metabolites. The metabolite profiling of 27 bioactive compounds was further investigated by docking analysis for the potential inhibition of the NF-κB pathway, which has an important function in inflammation and wound repair. The compounds eurobenzophenone A (7), aspernolide D (16), asperphenalenone A (23), aspergilate D (15), kodaistatin A (18), and versicolactone A (14) showed the highest binding affinity to the target protein with a pose score of −16.86, −14.65, −12.65, −12.45, −12.19, and −12.08 kcal/mol, respectively. Drug-likeness properties were also conducted. The findings suggest the potential wound healing properties of *A. terreus* as a source for lead therapeutic candidate discovery.

## 1. Introduction

Wounds are a common major health problem that affects the health sectors of many countries. Wound healing is a dynamic, complex, vital physiological process for tissue repair and restoration of skin integrity. Inflammation, tissue formation, and regeneration, as well as angiogenesis, are well-coordinated steps for wound healing [1]. Chronic inflammation has largely been evidenced to retard wound healing through disruption of the normal repair processes. The wound becomes chronic, with a prolonged inflammatory stage leading to retarding the proliferative and remodeling phases, which play a major role in healing. In addition, bacterial infections can be attributed to chronic processes. Recently, anti-inflammatory and antibacterial agents have been developed for resolving chronic inflammation and facilitating wound healing [2].

The exploration of natural products has been historically known as a rich source of drug development, in which fungi play a pivotal role in providing a diverse array of novel bioactive compounds. Fungi are known to possess significant biological activities, such as antimicrobial, anticancer, and immunosuppressive properties [3,4].

*Aspergillus terreus* is a filamentous fungus found as an endophyte or in soil-decaying vegetation. The genus *Aspergillus* is known for its bioactive secondary metabolite production ability. These metabolites have diverse biological activities and potential applications in the fields of medicine and agriculture. Among the numerous species within the genus, *A. terreus* is particularly notable for its production of terrein, lovastatin, and other biologically active compounds that have been extensively studied for their therapeutic potential. Notably, terrein, a major metabolite of *A. terreus*, has demonstrated promising strong anticancer activities by targeting multiple pathways, anti-inflammatory, and antimicrobial properties [5,6].

Additionally, lovastatin, another metabolite of *A. terreus*, was the first statin to be approved for clinical use and is widely recognized for its cholesterol-lowering effects and the treatment of hypercholesterolemia, emphasizing the therapeutic potential of fungal metabolites and their promising significance as reservoirs of bioactive compounds [7].

The identification and characterization of fungal metabolites have been greatly enhanced by advances in analytical techniques, such as liquid chromatography-mass spectrometry (LC-MS/MS), enabling the identification and characterization of a wide range of compounds with potential applications. The technique’s ability to perform high-throughput screening of fungal extracts has accelerated the pace of drug discovery, allowing researchers to efficiently identify promising lead compounds for further development [8,9,10].

The therapeutic potential of *A. terreus* extends beyond its well-known metabolites such as terrein and lovastatin. Recent studies have highlighted its significant antibacterial and cytotoxic effects, as well as its anti-inflammatory and antioxidant activities [11].

Molecular docking has become a cornerstone in drug discovery, providing a valuable tool for identifying and optimizing drug candidates. By integrating computational techniques, it enables faster and more cost-effective drug development, offering new possibilities for treating diseases. For instance, recent studies have explored the antiviral activity of *A. terreus* metabolites against SARS-CoV-2, identifying chaetominine as a promising inhibitor of the viral papain-like protease (PLpro). Such findings demonstrate the utility of molecular docking in identifying new therapeutic applications for fungal metabolites and guiding the design of novel drugs [6].

In the current study, we extended the investigation of the interesting fungus *A. terreus*, establishing promising anti-candidal, anti-staphylococcal, and antioxidant potency [12] by profiling its LC-MS/MS fingerprint and exploring its wound healing potential and molecular docking analysis of tentatively identified compounds. Furthermore, the integration of molecular docking simulations highlights the specific bioactivity of *A. terreus* metabolites in modulating key inflammatory pathways, an approach that is currently underexplored for selected metabolites. These findings introduce *A. terreus* as a promising source of novel therapeutic agents, particularly for applications in burn wound healing and inflammation-related disorders.

This work not only advances the field by uncovering new bioactive compounds but also demonstrates the utility of different research approaches, combining experimental and computational methods to identify and validate the therapeutic activity. This study’s findings pave the way for further research on bioassay-guided fractionation and clinical applications of these metabolites.

## 2. Materials and Methods

### 2.1. Study Fungus

*Aspergillus terreus* AUMC15447 (GenBank accession no. OR064355) was obtained from the cultures collection within the Mycology Laboratory, Department of Botany and Microbiology at the Faculty of Science, Assiut Branch, Al-Azhar University, Egypt [12].

### 2.2. Fermentation and Extraction

The fermentation process of the *A. terreus* strain was carried out on a solid rice medium, where approximately 1 mL of freshly prepared fungal spore suspension (~10^5^ CFUs/mL) was inoculated into fermentation flasks. The flasks were incubated in the dark for 30 days at 30 °C to promote fungal growth and secondary metabolite production [13]. After fermentation, the medium was extracted three times using ethyl acetate (El Nasr Company for Pharmaceutical Chemicals, Cairo, Egypt) (EtOAc) [14]. The resulting extract was then filtered through anhydrous sodium sulfate (El Nasr Company for Pharmaceutical Chemicals, Cairo, Egypt) to remove moisture and dried using a vacuum rotary evaporator (Heidolph Scientific Products GmbH, Schwabach, Germany) at 45 °C to afford a dry viscus extract that was kept for further analysis or bioactivity testing.

### 2.3. In Vitro Cell Migration Assay

The Human Skin Fibroblast (HSF) cell line was cultured at Nawah Scientific Inc., Amsterdam, The Netherlands, Egypt in Dulbecco’s modified eagle medium (DMEM) supported with penicillin (100 units/mL), streptomycin (100 mg/mL), and heat-inactivated 10% fetal bovine serum. The cells were maintained at 37 °C in a humidified incubator with 5% CO_2_. For the wound scratch assay, HSF cells were inoculated at a density of 2 × 10^5^ cells per well in a 12-well coated plate and incubated in 5% fetal bovine serum-Dulbecco’s modified eagle medium (FBS-DMEM) overnight at 37 °C with 5% CO_2_. The following day, scratches by removing a section of cells in the monolayer culture by mechanically scraping the surface with a sterile pipette tip were created in the cell monolayer, and the wells were rinsed with phosphate-buffered saline (PBS). Control wells were given fresh medium, while the treatment group received media containing the *A. terreus* extract 100 µg/mL. Images of cell migration into the scratch area were captured at different time points using an inverted microscope (Leica Microsystem, Wetzlar, Germany), with the plate kept at 37 °C and 5% CO_2_ between imaging. The wound closure was quantified by measuring the scratch area at each time point using Mil ImageView software, version 3.7 [15]. Each experiment was performed in triplicate, and the data were expressed as mean ± standard deviation.

### 2.4. Assessment of In Vivo Burn Wound Healing Potential

A total of twenty-four eight-week-old adult male Sprague-Dawley rats (200 ± 10 g) were obtained from the animal house at the Faculty of Pharmacy (Boys), Al-Azhar University, Cairo, Egypt. The animals were housed in separate compartments in the recommended environmental conditions with a 12-h light/dark cycle and fed with a rodent pellet regimen manufactured by the Egyptian Company for Oil and Soap, Cairo, Egypt with water ad libitum. After a 2-week adaptation period, the rats were utilized for the evaluation experiments. The rats were anesthetized using an intraperitoneal injection (i.p.) of xylazine and ketamine at doses of 5 mg/kg and 50 mg/kg, respectively. The dorsal hair of the rats was first trimmed with clippers and then fully removed using a chamomile-enriched hair removal cream designed for sensitive skin. Burns were created using a 20 mm diameter aluminum punch (El Sharq Scientific Company, Cairo, Egypt) that had been heated in boiling water (100 °C) for 30 sec. The heated punch was applied to the dorsal skin on both sides of each rat for 7 s without pressure, resulting in two full-thick circular burns on each rat’s back. To prevent shock, the rats administered a balanced salt solution at a dose of 40 mg/kg body weight.

The rats were randomly separated into four groups, each with 6 rats: the control group (I), the group subjected to burn induction without treatment (II), the group subjected to burn induction followed up by local application of the standard drug mupirocin (2%) (Mupirax ointment, Global Napi, Egypt) (III), and the group subjected to burn induction followed up by local application of *A. terreus* extract 2% ointment to the burn area for 14 days (IV). On day 14, all rats were euthanized with an anesthetic overdose. The rat skin burn model in all groups was conducted through the rats’ anesthesia, dorsal hair removal, and induction of two circular burns (20 mm in diameter) on both sides of each rat’s back using an aluminum stamp heated to 100 °C by boiling in water for 30 s. The burn stamp was applied to the skin for 7 s without pressure following the reported procedures after immersion in room-temperature water. The wound closure rate was assessed for each rat using a digital caliper and photo documentation [16].

Weekly measurements of wound closure were conducted using a digital caliper to determine the extent of occlusion of the length of the burn diameter on both sides of each rat. The measurement was recorded as a numeric value. In addition, digital photographs of the wound were taken from a 10 cm distance using a 12-MP Samsung digital camera (Samsung, Beni Suef, Egypt) with a 3-inch LCD, keeping the lens parallel to the burn wound. The EOS 4000D DSLR camera used was manufactured by Canon Inc. in Tokyo, Japan.

### 2.5. Histopathological Assessment

The skin samples from normal and wounded rats were preserved. The process involved two steps: fixation and dehydration. Fixation included submerging tissues in 10% buffered formalin for 24 h, followed by a 30-min distilled water rinse. Dehydration used a sequence of alcohol solutions (70%, 90%, and 100%). After that, samples were cleared with xylene, impregnated with paraffin wax, and sectioned (4–5 µm) for staining as described by Suvarna et al. [17], using hematoxylin and eosin stains, to identify various tissue abnormalities.

### 2.6. Statistical Analysis

Statistical analysis was carried out using GraphPad Prism 10.2.3.403 software. Fisher’s test was utilized with a 95% confidence interval for one-way analysis of variance (ANOVA) and t test.

### 2.7. LC-ESI-MS/MS Profiling

The ethyl acetate extract of *A. terreus* was analyzed using the SCIEX Triple Quad 5500+ MS/MS system (Agilent Technologies, Waldbronn, Germany) equipped with an electrospray ionization (ESI) positive mode for ion detection. The experimental condition and tentative identification of compounds were conducted using the reported protocol used by Al Mousa et al. [18]. A blank control in the LC-MS/MS analysis was used, and the peaks of the blank were filtered and excluded using MSdial software version 4.92.

### 2.8. Molecular Docking Simulation

Docking simulations were conducted using AutoDock Vina version 1.2.0 to assess the constituent’s affinity to NF-κB (PDB ID: 1IKN) and analyze the interaction patterns between the ligands and the target obtained from the Protein Data Bank (http://www.rcsb.org, accessed on 20 September 2024). The docking was conducted following the reported procedure by Banaganapalli et al. [19]. The docking results were recorded, and the interaction profiles were visualized using BIOVIA Discovery Studio (v21.1.0.20298).

### 2.9. Structure Drug-like Properties

The tentatively identified compounds with drug-like properties were evaluated by the Swiss ADME online tool (http://www.swissadme.ch, accessed on 12 September 2024) to predict their molecular characteristics [20].

## 3. Results

### 3.1. In Vitro Cell Migration Assay

**Figure 1:**
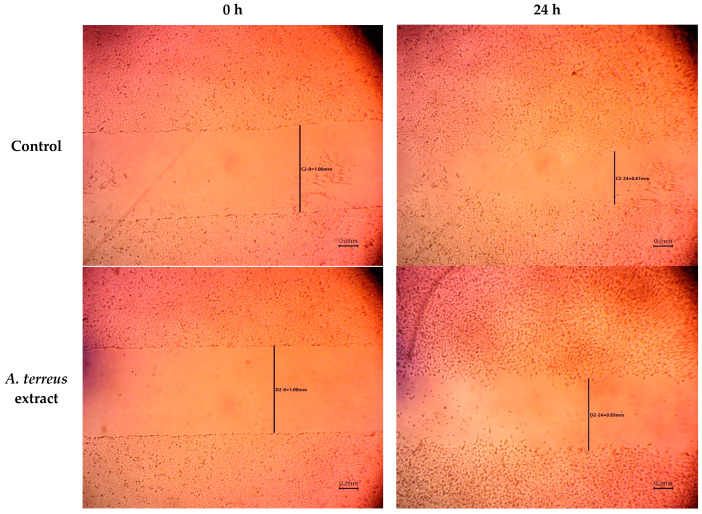

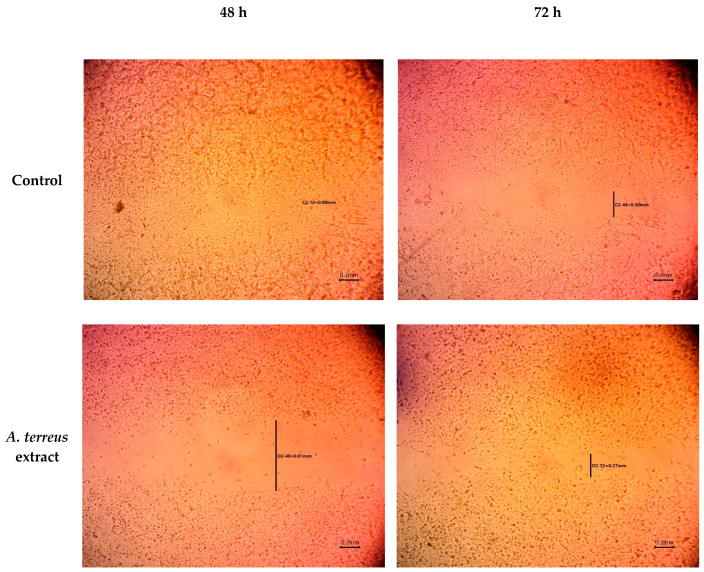
Wound width between edges of scratches over time using *A. terreus* extract treatment.

The *A. terreus* extract was assessed for its wound healing activity via the in vitro cell migration of the HSF cell line. As shown in Table 1, the drug displayed a moderate inhibition impact on cell migration with significantly slower wound closing within the treated group in comparison to the control, which in the tested time maintained some capacity for tissue repair (Figure 1). Also, the scratch wound width decreased as cell migration was induced. Slower closure may indicate a more controlled and organized migration of cells, leading to better tissue integrity and reduced scarring. Rapid closure can sometimes result in inadequate cellular signaling and incomplete repair mechanisms. The extract may promote a more balanced healing process that allows for appropriate remodeling of the extracellular matrix and tissue repair, which is crucial for optimal recovery.

### 3.2. Assessment of In Vivo Burn Wound Healing Potential

**Figure 2:**
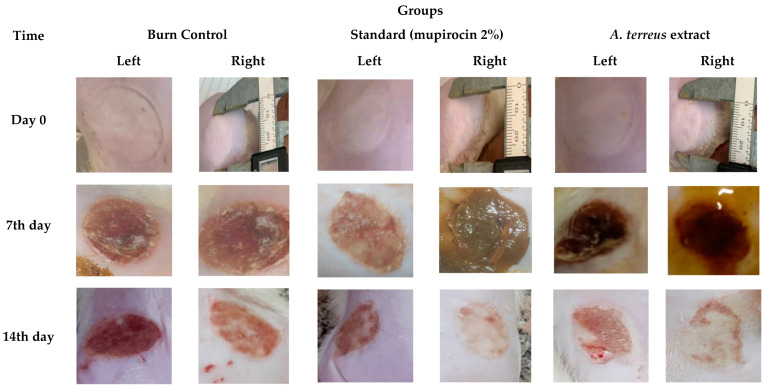
Photographs of burn injuries of dorsal tissues of rat groups over the experiment period.

After burn induction, pale lesions of circular scars were observed in treated as well as untreated animals. Blisters had appeared at 2 h, which palled at 6 h, and ruptured at 12 h, and blister size increased up to about a 20 mm diameter. In animals treated with *A. terreus* extract, wounds showed fluid leakage on the first day. On the third day, most of the wounds initially observed had slight oozing, but this subsided by the fifth day in all groups. Seven days later, a firm, red scab covered the healing wound that was already smaller in size but lasted longer, up until the 10th day in one untreated animal. In contrast, the treated animals had nearly healed wounds by Day 14, in which we could clearly see that the skin had regenerated over the area (Figure 2).

**Figure 3:**
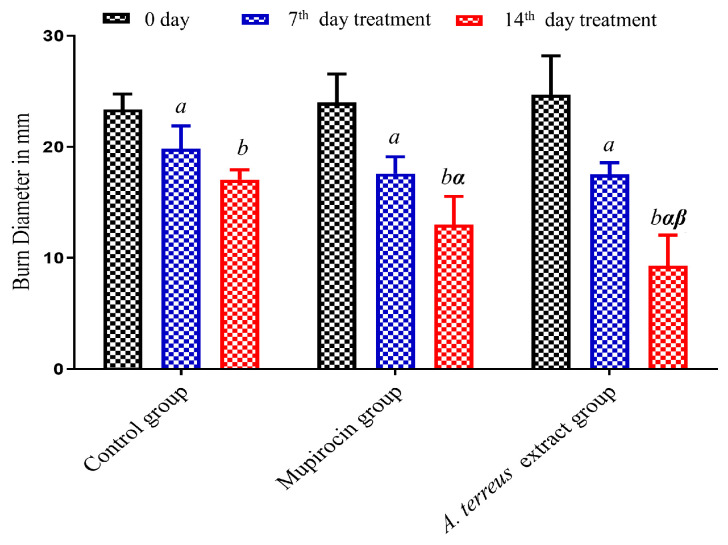
Burn wound’s contracture rate for the burn groups over time intervals. *^a^* Significantly different from Day 0 at *p* ≤ 0.05; *^b^* Significantly varied from 7th day at *p* ≤ 0.05; *^α^* Significantly varied from corresponding 7th day of control group at *p* ≤ 0.05; *^β^* Significantly different from corresponding 14th day of mupirocin group at *p* ≤ 0.05.

According to the results shown in Table 2 and Figure 3, wound healing was promoted by *A. terreus* extract, as the degree of wound closing induced by the burn group treated with mupirocin (2%) (Mupirax ointment) and the one treated with *A. terreus* extract had higher rates compared to the untreated group.

### 3.3. Histopathological Results

**Figure 4:**
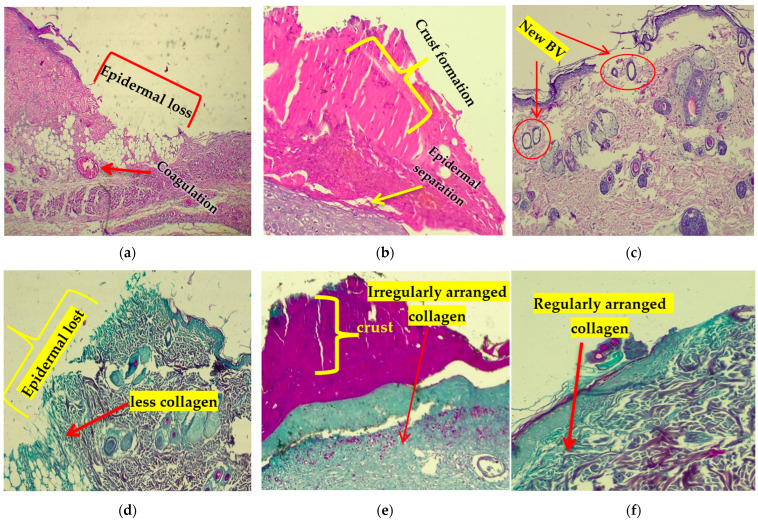
Photomicrograph of skin sections of (**a**) the burn group, showing burned skin histology and illustrated complete destruction of superficial skin layers and inflammatory changes with signs of coagulation in dermal layers, (**b**) the mupirocin-treated group, showing slight regeneration of the epidermis, crust formation with separation of dermal epidermal junction, and sever granulation tissue formation, and (**c**) the *A. terreus* extract-treated group, showing mild inflammation, regeneration of the epidermis, and less granulation tissue formation and with new vascularization (H&E × 400). (**d**) The burn group revealed complete destruction of superficial skin layers with fewer collagen fibers, (**e**) the mupirocin-treated group showed marked formation of irregularly arranged collagen fibers and granulation tissues, and (**f**) the *A. terreus* extract-treated group exhibited mild granulation tissue and regularly arranged collagen fiber formation (Masson trichrome × 400).

**Figure 5:**
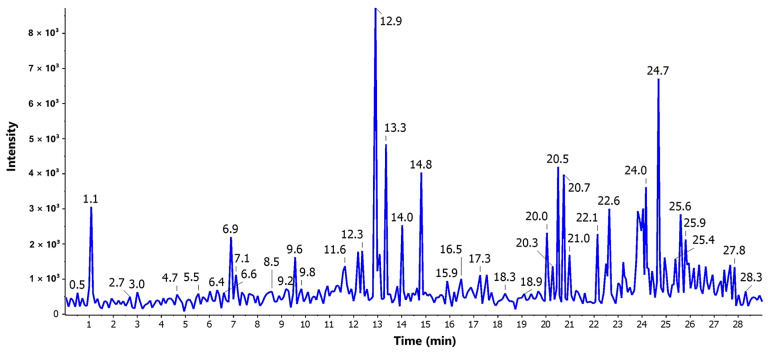
ESI-TIC chromatograms of the *A. terreus* EtOAc extract.

Histopathological examination of burn groups revealed destruction of superficial layers of the skin with inflammatory changes and sign of coagulation in dermal layers with less demonstrated collagen fibers. The mupirocin treated group exhibited moderate regeneration of epidermis, crust formation, and sever granulation tissue formation, while the *A. terreus* extract treated group revealed mild inflammation, regeneration of epidermis with less granulation tissue formation, and new vascularization (Figure 4 and Figure 5).

### 3.4. LC-ESI-MS/MS Profiling

The LC-MS-MS analysis of *A. terreus* crude extract was carried out for detection of secondary metabolites. Positive ionization mode was used to characterize the signals of corresponding compounds. The TIC of the *A. terreus* EtOAc extract was shown in Figure 5.

The components of the extract were analyzed to obtain characteristic fragment ions and a molecular formula. In addition, compounds isolated formerly from the genus *Aspergillus* were exploited as a tool for identifying the detected compounds by comparing the obtained molecular formula with the published data.

In total, 27 compounds were tentatively identified by analysis of their MS1 precursor ion, chemical formula, and fragmentation patterns compared with the CFMID online database. The results shown in Table 3 are arranged according to identified compounds’ retention times.

The mass spectrometric analysis of compounds extracted from various *Aspergillus* species reveals a striking diversity in molecular structures and fragmentation patterns, highlighting the complexity inherent in fungal metabolites. For instance, 11-Methyl-11-hydroxyldodecanoic acid amide (C_13_H_27_NO_2_) exhibits a primary ion at *m/z* 230.19, corresponding to [M + H]^+^, with significant fragments at *m/z* 213 and 171, suggesting dehydration and a possible McLafferty rearrangement. Likewise, 2,5-Dimethylresorcinol (C_8_H_10_O_2_) displays an [M + H]^+^ ion at *m/z* 139.08, which fragments to *m/z* 123 and 111, likely due to the loss of CH_3_ and a retro Diels-Alder reaction involving the aromatic ring. Further, 3-Hydroxy-2,5-toluquinone (C_7_H_6_O_3_) undergoes fragmentation at *m/z* 139.07 to yield *m/z* 121 and 111, attributed to CO loss and potential ring contractions.

In contrast, asnovolin B (C_26_H_38_O_7_) shows an [M + H]^+^ ion at *m/z* 463.27 with prominent fragments at *m/z* 445 and 403, indicative of sequential losses of H_2_O and CO_2_, likely through retro Diels-Alder reactions. Asperdemin (C_21_H_28_O_7_), with an ion at *m/z* 393.20, fragments to *m/z* 375 and 335, suggesting losses of H_2_O and CO_2_, with the McLafferty rearrangement contributing to the observed fragmentation pattern. Similarly, aspergilate D (C_14_H_18_O_7_) fragments from *m/z* 299.11 to *m/z* 281 and 223, indicative of dehydration and the loss of acetic acid, typical for esters. Aspergilazine A (C_20_H_20_N_2_O_8_S), with its [M + H]^+^ ion at *m/z* 449.10, shows fragments at *m/z* 431 and 393, which may result from sulfoxide group cleavage and subsequent rearrangements.

Further analysis reveals aspernolide D (C_24_H_26_O_9_), with an ion at *m/z* 459.17, fragments to *m/z* 441 and 401, indicating loss of H_2_O and rearrangements involving the lactone ring. Asperphenalenone A (C_35_H_44_O_7_) exhibits fragmentation at *m/z* 577.31 to *m/z* 559 and 545, likely through retro Diels-Alder reactions affecting the phenalenone moiety. Meanwhile, aspersclerolide B (C_8_H_12_O_5_), with a molecular ion at *m/z* 189.05, shows fragmentation to *m/z* 171 and 153, suggesting dehydration and possible lactone cleavage. Aspilactonol A (C_9_H_14_O_3_) reveals an ion at *m/z* 171.10, fragmenting to *m/z* 153 and 125, indicative of water loss and potential McLafferty rearrangement.

Additionally, banksialactone C (C_12_H_14_O_5_), with a molecular ion at *m/z* 239.03, fragments to *m/z* 221 and 193, likely due to water and CO loss, characteristic of lactones. Eurobenzophenone A (C_19_H_18_O_11_) displays an [M + H]^+^ ion at *m/z* 423.01, with fragments at *m/z* 387 and 329, indicative of sugar moiety loss and subsequent ring contractions. Flavicerebroside B (C_43_H_79_NO_9_) shows fragmentation at *m/z* 754.38 to *m/z* 736 and 718, likely through dehydration and fragmentation of the long aliphatic chain. Kodaistatin A (C_35_H_34_O_11_), with an ion at *m/z* 631.07, shows fragments at *m/z* 613 and 603, possibly due to retro Diels-Alder reactions and CO_2_ loss.

The compound methyl (Z)-4-{[(Z)-1-(hydroxymethyl)-2-phenyl-1-ethenyl] amino}-4-oxo-2-butenoate (C_14_H_15_NO_4_) shows an ion at *m/z* 262.16 that fragments to *m/z* 244 and 216, suggesting water loss and possible McLafferty rearrangement. Ochraceopone E (C_22_H_30_O_6_) exhibits an [M + H]^+^ ion at *m/z* 391.21, with fragments at *m/z* 356 and 251, likely due to ester cleavage and subsequent rearrangement. Pre-aurantiamine (C_11_H_14_N_4_O_2_) shows fragmentation at *m/z* 235.12 to *m/z* 219 and 191, indicative of amide bond cleavage and ring contractions. In a similar vein, pre-sclerotiotide F (C_20_H_30_N_4_O_5_) displays an ion at *m/z* 407.22 that fragments to *m/z* 389 and 295, suggesting water loss and possible McLafferty rearrangement.

Further, pyranterrone A/B (C_15_H_15_NO_4_) has an ion at *m/z* 274.07, which fragments to *m/z* 256 and 232, likely due to water loss and subsequent rearrangements. Terreic acid (C_7_H_6_O_4_) shows fragmentation at *m/z* 155.05 to *m/z* 137 and 127, suggesting CO loss and possible ring contractions. Terreinlactone B (C_8_H_10_O_2_) displays an ion at *m/z* 139.07 that fragments to *m/z* 121 and 111, indicative of retro Diels-Alder reactions involving the lactone ring. Terreprenphenol C (C_12_H_14_O_3_) has an ion at *m/z* 207.11 that fragments to *m/z* 189 and 171, likely due to water and CO loss. Lastly, terretonin D (C_26_H_34_O_8_), with an [M + H]^+^ ion at *m/z* 475.23, fragments to *m/z* 219 and 137, indicative of ester cleavage and subsequent rearrangements, while tropolactone B (C_28_H_36_O_8_) exhibits an ion at *m/z* 501.26, which fragments to *m/z* 483 and 469, likely due to water loss and possible retro Diels-Alder reactions. The data demonstrates the complicated biochemistry of Aspergillus species and its metabolites, indicating interesting opportunities for future research in natural product chemistry.

### 3.5. Molecular Docking Simulation and Drug-like Properties

The tentatively identified compounds from *A. terreus* extract were evaluated for their binding affinity to NF-κB as inhibitors. The results in Table 4 revealed that most compounds showed a promising affinity to the target in comparison to (E)-2-Fluoro-4ʹ-methoxystilbene as a standard inhibitor to NF-κB. The results revealed that the pose scores of the compounds ranged from −6.82 to −16.86 kcal/mol. The compounds eurobenzophenone A (7), aspernolide D (16), asperphenalenone A (23), aspergilate D (15), kodaistatin A (18), and versicolactone A (14) showed highest binding affinity to the target protein with pose scores of −16.86, −14.65, −12.65, −12.45, −12.19, and −12.08 kcal/mol, respectively. The analysis of various compounds’ physicochemical properties highlights their potential as drug candidates, focusing on Lipinski’s Rule of Five and bioavailability scores. Molecular weights range from 138.12 to 754.09 g/mol, with most compounds under 500 g/mol, indicating better permeability and oral bioavailability, though flavicerebroside B exceeds this limit, potentially hindering its effectiveness. Some compounds, such as eurobenzophenone A and asperphenalenone A, feature a high number of heavy and aromatic atoms, enhancing interactions but risking solubility issues. The number of rotatable bonds balances flexibility and rigidity, favoring bioactivity, while compounds with high hydrogen bond donors and acceptors may strengthen binding affinity at the cost of membrane permeability. TPSA values below 140 Å^2^ are generally favorable for absorption, with most compounds meeting this criterion, except for eurobenzophenone A and aspergillazine A. Most compounds adhere to Lipinski’s guidelines, but eurobenzophenone A and kodaistatin A exhibit multiple violations, posing challenges for oral activity. Bioavailability scores range from 0.11 to 0.85, with terreic acid and 3-hydroxy-2,5-toluquinone showing the highest scores (0.85), indicating strong potential for development. Overall, while many compounds exhibit promising characteristics, challenges such as high molecular weight, excessive aromaticity, and Lipinski’s rule violations need to be addressed to optimize drug-likeness and bioavailability.

However, the compounds eurobenzophenone A (7), kodaistatin A (18), and asperphenalenone A (23), which had the highest scores, showed violations against the drug-likeness Rule of Five (Table 5). Aspergilate D (15), versicolactone A (14), and aspernolide D (16) met all the criteria of Lipinski’s rule with promising bioavailability scores.

**Figure 6:**
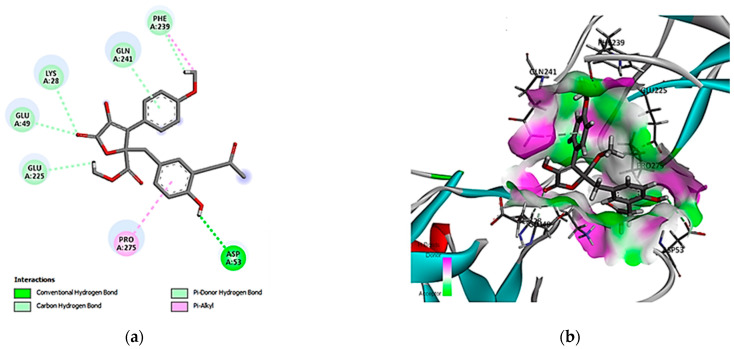
Two- and three-dimensional interactions complex of compound (14) with the NF-κB target. (**a**) Two-dimensional interactions of ligand with the active site and (**b**) three-dimensional interactions of ligand with the active site.

The interaction diagram (Figure 6) shows that versicolactone A (14) interacted with seven amino acid residues in the NF-κB-active site. Specifically, the compound interacted through conventional hydrogen bonding with Asp A53, indicating strong interactions that might contribute to the compound’s affinity, together with the carbon-hydrogen bond with Phe A239, Gln A241, Lys A28, Glu A49, and Glu A225 amino acid residues. Additionally, hydrophobic contacts were formed, which stabilized the ligand through non-polar interactions.

**Figure 7:**
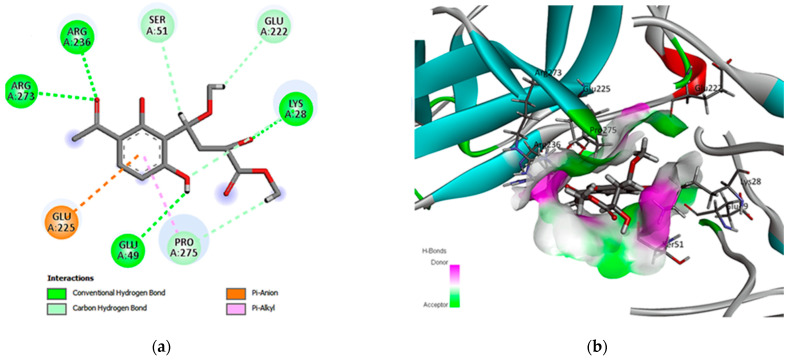
Two- and three-dimensional interactions complex of compound (15) with the NF-κB target. (**a**) Two-dimensional interactions of the ligand with the active site and (**b**) three-dimensional interactions of the ligand with the active site.

Moreover, for the compound aspergilate D (15), the interaction identified four amino acid residues involved in hydrogen-bonding interactions with Arg A236, Arg A273, Glu A49, and Lys A28 amino acids, stabilizing the ligand affinity to NF-κB (Figure 7).

**Figure 8:**
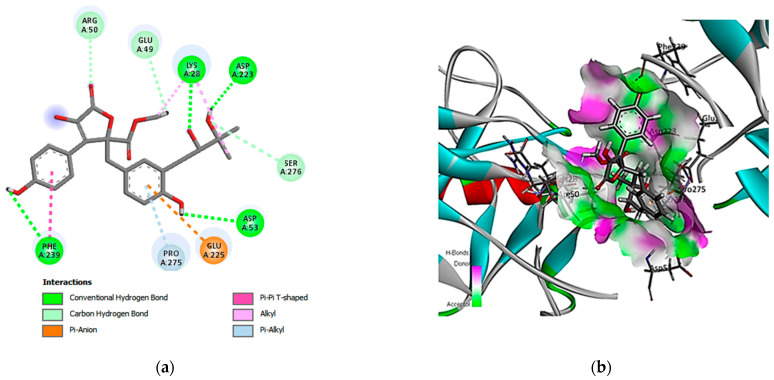
Two- and three-dimensional interactions complex of compound (16) with the NF-κB target. (**a**) Two-dimensional interactions of the ligand with the active site and (**b**) three-dimensional interactions of the ligand with the active site.

Finally, aspernolide D (16) exhibited interactions with nine amino acid residues, in which four were conventional hydrogen bonds (Lys A28, Asp A223, Asp A53, and Phe A239), three carbon hydrogen bonds (Arg A50, Glu A49, and Ser A276), and pi-anion, pi-pi, alkyl, and pi alkyl interactions, indicating strong interactions with these residues in the binding pocket (Figure 8).

## 4. Discussion

Burn injuries are considered a major health concern worldwide. Conventional burn treatment includes the use of dressings and some topical agents, which facilitate the healing of wounds and prevent bacterial infection of the injured wound [16]. The use of natural products provides many approved formulations with safe and effective treatments for burns and wounds. The current study provides an evaluation of the wound healing potential of metabolites derived from *A. terreus* by combined in vitro, in vivo, and in silico studies, offering valuable insights into the bioactivity of *A. terreus* extract as a burn wound healing lead source.

The evaluation of the wound healing potential of *A. terreus* extract conducted on HSF cells via scratch assay showed moderate promotion of cell migration, suggesting that the *A. terreus* extract did not promote rapid wound closure in the early phases of healing but retained the capacity to support tissue repair compared to the control. One possible explanation for the slower wound closure rate in vitro is that the extract might induce a controlled wound healing process, reducing excessive fibroblast proliferation which can lead to scarring.

Jonkman et al. [48] reported that the scratch cell line wound healing assay is considered as a standard in vitro technique for examining cell migration in two dimensions, where it is ordinarily encompassed in diverse pathological disorders.

In addition to the in vitro findings, the rat skin models’ burn treatment showed that local application of the *A. terreus* extract into burn wounds markedly enhanced wound healing and skin regeneration. The macroscopic observations and wound closure rates indicated that the *A. terreus* extract significantly enhanced wound healing when compared to the untreated group. By the 14th day, the burn diameter of wounds treated with the extract was reduced to 9.60 mm, significantly improved when compared to both the control group (16.78 mm) and the mupirocin-treated group (12.91 mm). These results could be attributed to the combined antibacterial and anti-inflammatory properties of the metabolites found in *A. terreus*. The ability to prevent bacterial infection, coupled with the modulation of inflammatory responses, played a critical role in facilitating faster wound closure, which was observed in this study. Additionally, the reduction of pro-inflammatory mediators, as previously reported in fungal secondary metabolites, may aid in the proper progression of the proliferative and remodeling phases of wound healing. These findings are consistent with a previous study on wound healing in adult Wistar rats conducted by Sreedevi et al. [49], which used bioactive fraction isolated from *Aspergillus terreus* intracellular pigment and established significant wound closure for *A. terreus* fraction (eight days) compared to boric acid (fourteen days). Salem et al. [50] reported a significant, promising wound healing efficiency and promoted wound contracture of EtOAc extract from endophytic *Paecilomyces* sp. AUMC15510 on earthworms, *Lumbricus castaneus*, after five days.

Effective wound healing involves a coordinated sequence of three overlapping phases: inflammation, proliferation, and remodeling [51]. The study findings revealed that the application of *A. terreus* extract significantly enhanced the wound healing process. By Day 14, the extract-treated group exhibited remarkable improvements (*p* < 0.001) compared to the untreated group. Further, the histopathological examination supported the results, showing improvement in collagen formation and deposition, vascularization, epithelial repair, and regeneration more than in the control group. *Aspergillus terreus* extract plays a crucial role in all stages of wound healing. The histopathological inspection of wounded adult Wistar rats treated with *A. terreus* bioactive fraction displayed the emigration of inflammatory cells, fibroblasts, and revascularization at the wounding area, accompanied by a healthy cellular structure and thick cell boundaries [49]. Salem et al. [50] reported that wounded earthworms treated with *Paecilomyces* sp. EtOAc extract retrieved their tissue’s normal structure on the fifth day.

After treatment with *A. terreus* extract, there was a noticeable acceleration in wound healing indicators, as re-epithelialization and thickness of regenerated epidermis promote regeneration following burn injury [52]. The investigation results revealed that *A. terreus* extract could promote the production of collagen and fibroblast production, vascularization, and anti-inflammatory properties. Collectively, the resulting data suggest that *A. terreus* extract contributes to enhanced wound healing outcomes.

For identification of possible active compounds, the LC-MS-MS analysis of *A. terreus* extract was conducted and led to tentative identification of 27 compounds. The anti-inflammatory properties of the identified metabolites may have played a role in reducing the chronic inflammation that is commonly associated with burn wounds, thereby supporting proper wound healing by inhibiting the NF-κB pathway signaling. NF-κB possesses a significant role in regulating immune response, inflammation, cell growth, and survival. The release of NF-κB promotes gene expression of pro-inflammatory cytokines and proteases such as MMP. However, excessive secretion of inflammatory mediators can lead to chronic wounds [53]. The docking studies targeted the NF-κB protein complex, a key regulator of inflammation. Aspergilate D (15), versicolactone A (14), and aspernolide D (16) showed high binding affinities, exhibiting the highest docking score and meeting the drug-likeness criteria of Lipinski’s rule. The in silico results complement the in vivo findings, as reduced inflammation is likely a key factor in the observed improved wound closure rates in treated animals

It is noteworthy that aspernolide A, a butyrolactone purified from endophytic *Cladosporium cladosporioides*, inhibited, in time- and dose-dependent modes, the proliferation of the human laryngeal carcinoma cell lines [54], which could illustrate the ceasing of wound closure in our study since aspernolide D was detected in the present investigation. However, the presence of other compounds could explain the wound healing potency of *A. terreus* extract. Moreover, Camptothecin derived from *A. terreus* exerted wound healing suppression on UO-31 tumor cells [55] and MCF-7 tumor cell scratches [56]. On the other hand, aspernolide C isolated from soft coral endophytic *A. terreus* exhibited slight cytotoxicity against five tested carcinogenic cell lines [57].

## 5. Conclusions

This study demonstrates the wound healing potential of *Aspergillus terreus* metabolites, as evidenced by a combination of in vitro, in vivo, and in silico studies. The metabolite profiling revealed a diverse array of bioactive compounds, several of which were shown to effectively promote wound healing by reducing inflammation and facilitating tissue repair. The results of molecular docking studies support the potential of these metabolites in targeting key inflammatory pathways with promising drug-likeness and bioavailability. These findings could serve as insight into further research targeting therapeutic applications of *A. terreus* metabolites, particularly in wound healing and inflammation-related diseases. Our future work will focus on the bioassay-guided fractionation and isolation of targeted compounds and studying their activity to validate their biological activities and explore their therapeutic potential through detailed mechanistic studies. This will elucidate the pathways through which these compounds exert their wound healing and anti-inflammatory effects, in addition to exploring the synergistic effects of these compounds with existing wound healing agents, which might offer new strategies for enhancing treatment outcomes. Overall, the therapeutic potential of *A. terreus* as a source of bioactive compounds warrants further investigation to fully harness its benefits in medical applications.

## Figures and Tables

**Table 1:** Wound width between the edges of the scratches treated with *A. terreus* extract over time.

Time (h)	Wound Width (mm)	
	Control	*A. terreus* Extract
0	0.99 ± 0.027	1.02 ± 0.040
24	0.60 ± 0.015	0.84 ± 0.006
48	0.31 ± 0.012	0.82 ± 0.006
72	0.00 ± 0.00	0.30 ± 0.0252

Data were presented as the means of three replicates (mean ± SD).

**Table 2:** The diameter of the burn wound’s closure rate for the burn groups over two time intervals.

Time (day)	Groups–Burn Diameter (mm)		
	Control	Mupirocin	*A. terreus* Extract
Day 0	23.13 ± 0.55	24.08 ± 0.93	24.41 ± 1.30
7th day	20.21 ± 0.82 *^a^*	17.08 ± 0.75 *^a^*	16.95 ± 0.68 *^a^*
14th day	16.78 ± 0.41 *^b^*	12.91 ± 0.93 *^b^*^,^ *^α^*	9.60 ± 1.05 *^b^*^,^ *^α^*^,^ *^β^*

Data are presented as means ± SEM, n = 6. *^a^* Significantly different from Day 0 at *p* ≤ 0.05; *^b^* Significantly varied from 7th day at *p* ≤ 0.05; *^α^* Significantly varied from corresponding 7th day of control group at *p* ≤ 0.05; *^β^* Significantly different from corresponding 14th day of mupirocin group at *p* ≤ 0.05; using one-way ANOVA.

**Table 3:** List of tentatively identified compounds from *A. terreus* extract.

No.	t_R_	M + H	MF	MS2	Compound Name	Reported Source	Ref.
1	5.65	155.05	C_7_H_6_O_4_	137, 127	Terreic acid	*A. terreus*	[21]
2	8.57	139.07	C_7_H_6_O_3_	121, 111	3-hydroxy-2,5-toluquinone	*A. fumigatus*	[22]
3	8.94	262.16	C_14_H_15_NO_4_	244, 216, 200, 132, 115	Methyl (Z)-4-[(Z)-1-(hydroxymethyl)-2-phenyl-1-ethenyl] amino-4-oxo-2-butenoate	*A. niger*	[23]
4	10.17	171.10	C_9_H_14_O_3_	153, 125, 111, 107, 103	Aspilactonol A	*Aspergillus* sp. 16-02-1	[24]
5	10.59	139.07	C_8_H_10_O_2_	121, 111, 109	Terreinlactone B	*A. terreus*	[25]
6	10.84	239.03	C_12_H_14_O_5_	221, 193, 177, 163, 149, 135	Banksialactone C	*A. banksianus*	[26]
7	11.58	423.01	C_19_H_18_O_11_	387, 329, 313, 287	Eurobenzophenone A	*A. europaeus* WZXY-SX-4-1	[27]
8	11.97	139.08	C_8_H_10_O_2_	123, 121, 111, 103	2,5-Dimethylresorcinol	*A. nidulans*	[28]
9	13.02	230.19	C_13_H_27_NO_2_	213, 212, 171, 167, 137, 129	11-methyl-11-hydroxyldodecanoic acid amide	*A. fumigatus* JRJ111048	[29]
10	13.85	274.07	C_15_H_15_NO_4_	256, 232, 218, 200	Pyranterrone A/B	*A. terreus*	[30]
11	14.20	189.05	C_8_H_12_O_5_	171, 153, 141, 115, 105	Aspersclerolide B	*A. sclerotiorum*	[31]
12	14.65	449.10	C_20_H_20_N_2_O_8_S	431, 393, 363, 315, 285, 212	Aspergillazine A	*A. unilateralis*	[32]
13	17.37	235.12	C_11_H_14_N_4_O_2_	219, 191, 179, 175, 219, 191, 179, 175, 135, 119	Pre-aurantiamine	*A. aculeatus* CRI322-03	[33]
14	17.57	427.14	C_23_H_22_O_8_	325, 295	Versicolactone A	*A. versicolor*	[34]
15	18.03	299.11	C_14_H_18_O_7_	281, 267, 223, 197, 167	Aspergilate D	*Aspergillus* sp.	[35]
16	18.05	459.17	C_24_H_26_O_9_	441, 401, 357, 221	Aspernolide D	*A. terreus* RCBC1002	[36]
17	18.80	207.11	C_12_H_14_O_3_	189, 171, 149, 135, 123, 121,105	Terreprenphenol C	*A. terreus* EN-539	[37]
18	19.11	631.08	C_35_H_34_O_11_	613, 603, 585, 575, 557, 529, 463	Kodaistatin A	*A. terreus* DSM 11247	[38]
19	20.29	393.20	C_21_H_28_O_7_	375, 335, 323, 281, 267, 253	Asperdemin	*A. versicolor*	[39]
20	20.45	463.27	C_26_H_38_O_7_	445, 403, 389, 359, 305, 223, 207	Asnovolin B	*A. novofumigatus* CBS117520	[40]
21	22.87	391.25	C_23_H_34_O_5_	373, 363, 356,253, 237, 225	Ustusolate A	*A. ustus* 094102	[41]
22	23.61	501.26	C_28_H_36_O_8_	483, 469, 379, 273	Tropolactone B	*Aspergillus* sp.	[42]
23	23.73	577.31	C_35_H_44_O_7_	559, 545, 531, 491, 451, 449, 447, 437, 421, 285	Asperphenalenone A	*Aspergillus* sp. CPCC 400735	[43]
24	24.64	475.23	C_26_H_34_O_8_	219, 137, 131, 121	Terretonin D	*A. terreus*	[44]
25	25.64	407.22	C_20_H_30_N_4_O_5_	389, 295, 282	Pre-sclerotiotide F	*A. insulicola*	[45]
26	25.69	754.38	C_43_H_79_NO_9_	736, 718, 574, 556, 294	Flavicerebroside B	*A. flavipes*	[46]
27	27.55	391.21	C_22_H_30_O_6_	356, 251, 237, 223, 149, 121	Ochraceopone E	*A. ochraceopetaliformis* SCSIO 05702	[47]

**Table 4:** Docking scores of identified compounds from *A. terreus* extract against NF-κB (PDB ID: 1IKN).

No.	Compound Name	Pose Score (kcal/mol)	No.	Compound Name	Pose Score (kcal/mol)
1	Terreic acid	−9.63	15	Aspergilate D	−12.45
2	3-hydroxy-2,5-toluquinone	−10.14	16	Aspernolide D	−14.65
3	Methyl (Z)-4-[(Z)-1-(hydroxymethyl)-2-phenyl-1-ethenyl] amino-4-oxo-2-butenoate	−10.58	17	Terreprenphenol C	−9.53
4	Aspilactonol A	−8.64	18	Kodaistatin A	−12.19
5	Terreinlactone B	−6.82	19	Asperdemin	−9.62
6	Banksialactone C	−11.33	20	Asnovolin B	−8.11
7	Eurobenzophenone A	−16.86	21	Ustusolate A	−8.41
8	2,5-Dimethylresorcinol	−8.71	22	Tropolactone B	−11.59
9	11-methyl-11-hydroxyldodecanoic acid amide	−9.59	23	Asperphenalenone A	−12.65
10	Pyranterrone A/B	−11.09	24	Terretonin D	−9.61
11	Aspersclerolide B	−11.18	25	Pre-sclerotiotide F	−8.64
12	Aspergillazine A	−9.86	26	Flavicerebroside B	−10.71
13	Pre-aurantiamine	−9.47	27	Ochraceopone E	−8.73
14	Versicolactone A	−12.08	28	(E)-2-Fluoro-4′-methoxystilbene	−8.24

**Table 5:** Drug-like properties of identified compounds from *A. terreus* extract.

No.	Compound Name	MW	Heavy Atoms	Aromatic Heavy Atoms	Rotatable Bonds	H-Bond Acceptors	H-Bond Donors	TPSA	Lipinski Violations	Bioavailability Score
1	Terreic acid	154.12	11	0	0	4	1	66.9	0	0.85
2	3-hydroxy-2,5-toluquinone	138.12	10	0	0	3	1	54.37	0	0.85
3	Methyl (Z)-4-[(Z)-1-(hydroxymethyl)-2-phenyl-1-ethenyl] amino-4-oxo-2-butenoate	261.27	19	6	7	4	2	75.63	0	0.55
4	Aspilactonol A	170.21	12	0	3	3	1	46.53	0	0.55
5	Terreinlactone B	138.16	10	0	1	2	0	26.3	0	0.55
6	Banksialactone C	238.24	17	6	0	5	3	86.99	0	0.55
7	Eurobenzophenone A	422.34	30	12	8	11	7	202.05	2	0.11
8	2,5-Dimethylresorcinol	138.16	10	6	0	2	2	40.46	0	0.55
9	11-methyl-11-hydroxyldodecanoic acid amide	229.36	16	0	10	2	2	63.32	0	0.55
10	Pyranterrone A/B	273.28	20	9	3	4	2	87.23	0	0.55
11	Aspersclerolide B	188.18	13	0	3	5	2	75.99	0	0.55
12	Aspergillazine A	448.45	31	10	5	9	4	164.79	0	0.55
13	Pre-aurantiamine	234.25	17	5	2	3	3	86.88	0	0.55
14	Versicolactone A	426.42	31	12	8	8	2	119.36	0	0.55
15	Aspergilate D	298.29	21	6	7	7	3	113.29	0	0.55
16	Aspernolide D	458.46	33	12	8	9	5	153.75	0	0.55
17	Terreprenphenol C	206.24	15	6	3	3	1	49.83	0	0.55
18	Kodaistatin A	630.64	46	12	10	11	6	198.89	3	0.11
19	Asperdemin	392.44	28	6	0	7	2	106.2	0	0.55
20	Asnovolin B	462.58	33	0	3	7	1	99.13	0	0.56
21	Ustusolate A	390.51	28	0	7	5	3	86.99	0	0.55
22	Tropolactone B	500.58	36	7	4	8	0	105.2	1	0.55
23	Asperphenalenone A	576.72	42	10	12	7	5	135.29	1	0.55
24	Terretonin D	474.54	34	0	2	8	1	124.04	0	0.55
25	Pre-sclerotiotide F	406.48	29	0	6	5	3	124.68	0	0.55
26	Flavicerebroside B	754.09	53	0	34	9	7	168.94	2	0.17
27	Ochraceopone E	390.47	28	6	0	6	2	96.97	0	0.55

## Data Availability

Data are contained within the article.
